# Correlation between an angiographic and a cardiac magnetic resonance score of myocardial jeopardy using standard and high-resolution perfusion sequences

**DOI:** 10.1186/1532-429X-13-S1-P89

**Published:** 2011-02-02

**Authors:** Geraint Morton, Masaki Ishida, Kalpa De Silva, Pierre Sicard, Amedeo Chiribiri, Andreas Schuster, Shazia Hussain, Matthias Paul, Divaka Perera, Eike Nagel

**Affiliations:** 1 King's College London, London, UK

## Objective

Evaluate the correlation between a Cardiac Magnetic Resonance (CMR) and an angiographic myocardial jeopardy score in coronary artery disease (CAD) using either a high-resolution or a standard perfusion sequence.

## Background

High temporal resolution required for CMR perfusion imaging results in compromised spatial resolution. However, recent advances, including the use of the advanced acceleration method k-t sensitivity encoding, allow myocardial perfusion imaging with unprecedented spatial resolution. The associated benefits remain to be fully described.

The BCIS-1 Myocardial Jeopardy Score (JS) quantifies the amount of myocardium in jeopardy from severe CAD (0= no jeopardy; 12 = maximum jeopardy). It accommodates patients with CABG and left main disease, is prognostically relevant and can easily be calculated by visual analysis of the X-ray coronary angiogram (XCA). Combined perfusion and scar CMR imaging should provide equivalent functional information on myocardial jeopardy. High-resolution perfusion imaging may improve the correlation between JS and CMR findings.

## Methods

40 consecutive study patients with known or suspected CAD underwent 1.5T CMR imaging and standard diagnostic XCA. CMR stress and rest perfusion imaging included 3 transverse slices every heartbeat using a standard TFE (st-TFE) sequence (17 patients) or a high-resolution kt balanced Turbo Field Echo (kt-TFE) sequence (23 patients). Typical acquired spatial resolution: 2.8 x 2.5 x 10mm and 1.7 x 1.9 x 10mm respectively. Cine and scar imaging were also performed.

Data analysis was blind. CMR perfusion and scar data were segmented using the standard 17-segment model excluding the apex. Each segment was subdivided into equal endo and epicardial sub-segments. Sub-segments were assigned as 3% of the total myocardium each and classified as normal, ischaemia or scar. Percentage myocardium affected by ischaemia or scar was then calculated.

## Results (tables 1 and 2)

**Table 1:** st-TFE patient characteristics.

Age (mean ± SD)	64±8 years
Coronary Artery Disease	82%
Male	71%
Hypertension	82%
Diabetes	41%
Previous CABG	0%
Previous PCI	6%

**Table 2:** kt-TFE patient characteristics

Age (mean ± SD)	67±11 years
Coronary Artery Disease	96%
Male	78%
Hypertension	61%
Diabetes	35%
Previous CABG	26%
Previous PCI	26%

**Figure 1:**
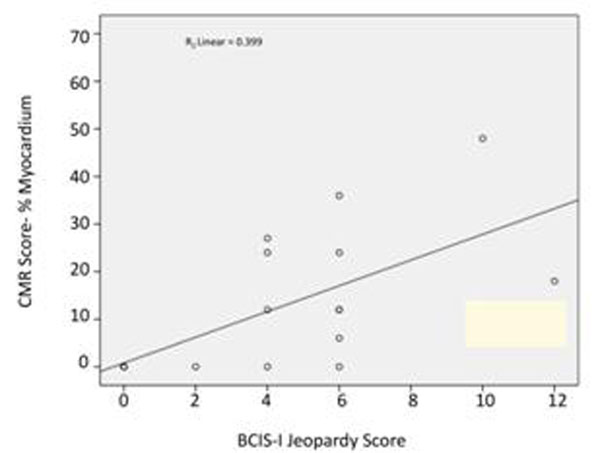
CMR Ischaemia/Scar Score versus JS st TFE perfusion cases

**Figure 2:**
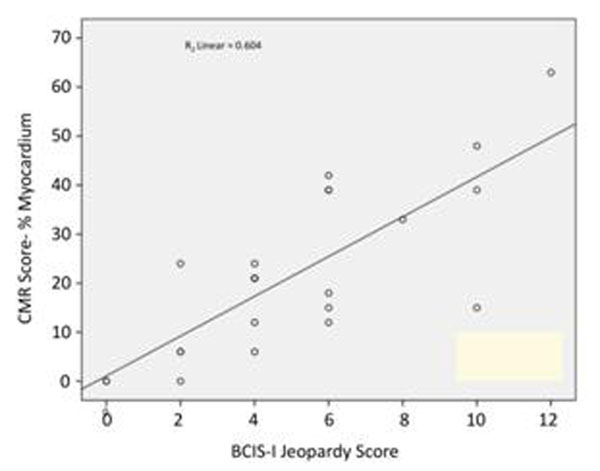
CMR Ischaemia/Scar Score versus JS kt TFE perfusion cases

There was a significant correlation between the JS and CMR score which was strongest in the high-resolution kt-TFE group; r=0.78, p=<0.0001 and appeared less strong in the st-TFE group; r=0.63, p=0.007 (figures 1 and 2).

## Conclusions

There is a significant correlation between the anatomic JS and a functional CMR ischaemia/scar score. This correlation seems to improve with the use of a high-resolution perfusion technique. Complete correlation between the two scores would not be expected for reasons such as difficulty predicting the haemodynamic effects of anatomically moderate disease or post-revascularization when scar may be subtended by patent vessels. However, the correlation is likely to be clinically useful and using a high-resolution perfusion sequence may therefore be superior for imaging patients with CAD.

